# Main trauma conference – the first day

**DOI:** 10.1186/1757-7241-20-S1-I2

**Published:** 2012-03-22

**Authors:** Kate Crewdson

**Affiliations:** 1 Bristol, UK

## 

The event organisers, Professor David Lockey and Dr Gareth Davies, both Pre-hospital Care Specialists at London’s Air Ambulance, as well as consultants in Anaesthesia and Intensive Care, and Emergency Medicine respectively, welcomed the delegates before proceedings got underway. The hosts had devised a diverse programme with a faculty of renowned subject experts from across the world. Each invited speaker had been challenged to answer 3 specific questions on their expert area with the aims of challenging dogma, airing contentious issues and encouraging excellence in pre-hospital care.

Major haemorrhage is a leading cause of death in trauma patients, and the first session of the day focussed on this important issue. Current data suggest 40% of trauma deaths may be attributed to uncontrolled haemorrhage, and 55% of trauma patients arriving at hospital are coagulopathic; 3% require massive transfusion. Dr Anne Weaver, a consultant at the London Air Ambulance, spoke on the topic of Massive Transfusion and the implementation of a ‘Code Red’ policy for management of massive haemorrhage, developed in the Major Trauma Centre (MTC) at which the London Air Ambulance is based. The trauma team leader declares Code Red if the systolic blood pressure is less than 90mmHg, a poor response to initial fluid resuscitation and suspected active haemorrhage. Pre-hospital Code Red activation provides an early pre-hospital alert to the receiving hospital ensuring packed red cells are available at the helipad on arrival of the aeromedical team; in addition the Level 1 cell salvage system is primed with blood, and fresh frozen plasma is requested from the laboratory. Furthermore Dr Weaver discussed the roles of prothrombin complex concentrate and tranexamic acid in the reduction of mortality from major haemorrhage. Dr Paul Wallman, a Manchester emergency medicine consultant, focussed on early reversal of warfarin in trauma patients found to be receiving oral anticoagulation. Whilst several guidelines in current clinical practice highlight the need for recognition of anticoagulated patients who sustain trauma, this does not always occur in a timely fashion. Early identification of this patient group, and subsequent treatment with vitamin K, prothrombin complex concentrate and fresh frozen plasma can improve outcome, and Dr Wallman was able to demonstrate this with personal data. The session was concluded by Professor Karim Brohi speaking on the topic of Damage Control Surgery, an eminent trauma surgeon, Professor Brohi was able to speak from firsthand experience. His talk centred around the pros and cons of damage control surgery and the triad of hypothermia, acidosis, and coagulopathy commonly associated with trauma.

The second session was entitled ‘Novel Thoughts’ which provided two excellent talks giving alternative views to current clinical doctrine. Professor Jonathan Benger discussed the routine use of cervical spine immobilisation in the management of trauma patients, highlighting the fact that there is no high quality research indicating spinal immobilisation benefits patients. Professor Benger argued that as significant injury is rare, most immobilisation is unnecessary. He questioned the rationale of immobilising patients ‘just in case’, or immobilising those patients walking on scene, or those who walk into the Emergency Department. Cervical spine immobilisation is not without complications, most significantly, it can make the airway more difficult to manage, and slow pre-hospital extrication and therefore delay the onset of definitive care. In conclusion, Professor Benger suggested that alert and cooperative patients should be clinically assessed and then managed appropriately; non-alert patients are at risk of cervical spine injury and should be immobilised. The final talk in this session was from Dr Emrys Kirkman a research scientist working with the UK military. He presented evidence on the potential time limitations of hypotensive resuscitation and discussed the concept of novel hybrid resuscitation where after a period of hypotensive resuscitation normotension is targeted to maintain organ perfusion.

In a keynote address Professor James Fawcett from Cambridge University talked about his work in the field of novel treatments for spinal cord injury. Many exciting new therapeutic possibilities were presented.

After lunch a session entirely devoted to different aspects of Head Injury took place. Dr Dean Kerslake, a trainee in Emergency Medicine and Intensive Care was able to give a first-hand account of his own experience of significant head injury. This was followed by Dr Alan Carson, a consultant neuropsychiatrist, providing insight into the Neuropsychiatric Complications of Trauma. The major depressive signs following traumatic brain injury were shown to be anxiety and irritability; up to 50% of patients will have cognitive problems and 50% will have mood problems. Mr Peter Hutchinson, a consultant neurosurgeon from Cambridge spoke on the management of raised intracranial pressure (ICP) following head injury and specifically the use of decompressive craniectomy, which has not been shown to be associated to improve morbidity or mortality as initially thought [1]. An update of the RESCUEicp study, a large multicentre study developed by the Academic Neurosurgical Division at Cambridge University, showed it to be nearing completion of recruitment. The study hypothesises that the application of decompressive craniectomy to head-injured patients with raised and refractory ICP results in improvement in outcome compared to optimal medical management. Thereafter, the content of the talk turned to medical management of head injury including hypothermia and the use of drugs such as progesterone, cyclosporin, and tranexamic acid, as well as multimodality monitoring of cerebral blood flow, pressure, oxygenation and metabolism, and microdialysis.

The final session of the day focussed on paediatric trauma. Dr Ffion Davies, a consultant in Emergency Medicine, who also trained in paediatrics, was asked to discuss whether adult trauma clinicians should care for injured children. Relatively few paediatric trauma centres exist, given the low (and declining) rate of paediatric trauma when compared to adults. Dr Davies suggested that the design of a trauma system for children should reflect the design of an adult system i.e. a pre-hospital phase, the first 24 hours, ongoing care, rehabilitation and trauma networks. The establishment of regional or supraregional advisory networks with a consultant in pre-hospital care and paediatrics available via telephone may help to improve quality of care and the accuracy of triage decisions. If a child is triaged to the nearest (adult) major trauma centre in order to expedite time to definitive care, secondary transfer by a specialist paediatric retrieval team to a paediatric centre may be required.

Dr Amber Young concluded the first day of the London Trauma Conference with a thorough and educational review of paediatric burns. Sadly this appears to be a problem that is increasing in severity, particularly in the under two years age category. Specifically, Dr Young was asked to address the issue of fluid management for burns patients, which remains controversial. This provided a very interesting focus to the talk, whilst traditional teaching has leaned towards high volume fluid resuscitation, this has a tendency to result in volume overload, which may be harmful. Dr Young suggested the concept of permissive hypovolaemia in the management of paediatric burns, moving away from the need to maintain normal cardiovascular and urinary parameters with fluids and inotropes. Permissive hypovolaemia is achieved using 2ml/kg/% burn and 80% of maintenance, with the use of diuretics if volume overload is suspected. Whilst adequate organ perfusion is obviously crucial, over resuscitation has been associated with respiratory compromise, nocosomial pneumonia, and increased length of critical care and hospital stay.
